# High hemoglobin levels are associated with decreased risk of diabetic retinopathy in Korean type 2 diabetes

**DOI:** 10.1038/s41598-018-23905-2

**Published:** 2018-04-03

**Authors:** Min-Kyung Lee, Kyung-Do Han, Jae-Hyuk Lee, Seo-Young Sohn, Jee-Sun Jeong, Mee-Kyoung Kim, Ki-Hyun Baek, Ki-Ho Song, Hyuk-Sang Kwon

**Affiliations:** 1 Division of Endocrinology and Metabolism, Department of Internal Medicine, Myongji hospital, Gyeonggi-do, Republic of Korea; 2 Department of Medical Statistics, College of Medicine, The Catholic University of Korea, Seoul, Republic of Korea; 3 Division of Endocrinology and Metabolism, Department of Internal Medicine, Yeouido St. Mary’s Hospital, College of Medicine, The Catholic University of Korea, Seoul, Republic of Korea

## Abstract

Anemia is an independent risk factor for the development of diabetic retinopathy (DR) in patients with type 2 diabetes mellitus (DM). Hemoglobin levels may also be associated with DR. We investigated the association between hemoglobin levels and DR risk. This cross-sectional, population-based study utilized data from 2,123 type 2 DM patients aged ≥30 years who participated in the Korea National Health and Nutrition Examination Survey from 2008 to 2012. Participants underwent an ophthalmic examination, including fundus photographs. A multiple logistic regression analysis was performed to evaluate the relationship between hemoglobin levels and DR risk. The mean hemoglobin levels in patients with and without DR were 13.76 ± 0.12 and 14.33 ± 0.05 g/dL, respectively, with anemia observed in 16.2 (2.4)% and 7.8 (0.8)%, respectively. A 19% decrease in DR risk was found with a 1.0-g/dL increase in hemoglobin level. DR risk exhibited a decreasing trend with increasing hemoglobin levels (*P* for trend <0.0001). The adjusted odds ratio of DR was significantly lower in the highest hemoglobin quartile. Our findings indicate that high hemoglobin levels are significantly related to a decreased DR risk in Korean type 2 diabetes.

## Introduction

Diabetes mellitus (DM) is one of the foremost public health issues worldwide that can lead to complications in many organ systems. The prevalence of DM in Korea has increased^1^ and its complications are becoming the major causes of morbidity and mortality^2^. Diabetic complications ultimately impact quality of life and mortality and are associated with increased medical costs^3^. Multifactorial treatment approaches including proper glycemic control, are critical in preventing diabetic complications.

Diabetic retinopathy (DR) is a frequent microvascular complication of DM that leads to severe vision loss^4^. It refers to progressive pathological alterations from mild nonproliferative abnormalities to moderate and severe nonproliferative DR (NPDR) to proliferative DR (PDR), which is characterized by abnormal changes in the retinal microvasculature that cause retinal nonperfusion, increased vasopermeability, and pathological intraocular proliferation of the retinal vessels^5^. Various factors are associated with the development and severity of DR including glycaemic control, DM duration, age, and albuminuria^6^.

Anemia is a common complication in patients with type 2 DM^7^ and is defined as hemoglobin level of <13 g/dL in men and <12 g/dL in women^8^. It is considered a risk factor for microvascular complications, including retinopathy, nephropathy, and neuropathy in diabetic patients^9,10^. Although the pathogenic mechanisms of DR remain unclear, several studies have suggested that anemia^11–13^ and hemoglobin levels^14–16^ may be linked to the development and progression of DR.

In this study, a cross-sectional study was conducted to evaluate the association between hemoglobin levels and the rate of anemia and the development and severity of DR in Korean type 2 diabetes.

## Results

### Clinical characteristics of the study population

Table 1 summarizes the baseline clinical characteristics of the study population based on DR status. Of the 2,123 participants (1,065 men and 1,058 women), 391 had DR and 1,732 did not. The patients with DR were older (60.1 ± 0.7 vs. 57.4 ± 0.4 years), had longer duration of DM (10.7 ± 0.5 vs. 4.5 ± 0.2 years), and had a higher prevalence of anemia [16.2 (2.4) vs. 7.8 (0.8)%] compared to those without DR. Significant differences were observed in the body mass index (BMI) (24.3 ± 0.3 vs. 25.5 ± 0.1 kg/m²), hemoglobin level (13.76 ± 0.12 vs. 14.33 ± 0.05 g/dL), glycated hemoglobin (HbA1c) level (8.1 ± 0.1 vs. 7.3 ± 0.1%), fasting plasma glucose (FPG) level (143 ± 1.4 vs. 159.5 ± 3 mmol/L), and estimated glomerular filtration rate (eGFR) (85.2 ± 1.3 vs. 91 ± 0.6 ml/min/1.73 m²) between patients with and without DR, respectively. The use of insulin therapy [15.6 (2.3) vs. 4.7 (0.7)%] and oral hypoglycaemic agent [85.8 (2.1) vs. 56.0 (1.4)%] were higher among patients with DR. Sex, total cholesterol, hypertension, metabolic syndrome, smoking status and drinking history did not significantly differ between the groups.

**Table 1:** Baseline characteristics of patients without or with DR.

	No DR (n = 1732)	DR (n = 391)	P value
Age, years	57.4 ± 0.4	60.1 ± 0.7	0.0009^*^
Sex (male), %	55.3(1.4)	54(3.2)	0.7061
Duration of diabetes, years	4.5 ± 0.2	10.7 ± 0.5	<0.0001^*^
Insulin therapy, %	4.7(0.7)	15.6(2.3)	<0.0001^*^
Oral hypoglycaemic agent, %	56.0(1.4)	85.8(2.1)	<0.0001^*^
BMI, kg/m²	25.5 ± 0.1	24.3 ± 0.2	<0.0001^*^
Waist circumference, cm	87.9 ± 0.3	85.9 ± 0.5	0.0005^*^
HbA1c, %	7.3 ± 0.1	8.1 ± 0.1	<0.0001^*^
FPG, mmol/L	143 ± 1.4	159.5 ± 3	<0.0001^*^
Total cholesterol, mg/dL	189.2 ± 1.3	186.2 ± 2.6	0.3071
GFR, ml/min/1.73 m²	91 ± 0.6	85.2 ± 1.3	0.0001^*^
Hemoglobin, g/dL	14.33 ± 0.05	13.76 ± 0.12	<0.0001^*^
Anemia, %	7.8(0.8)	16.2(2.4)	<0.0001^*^
Hypertension, %	58.8(1.6)	54.9(2.9)	0.2228
Metabolic syndrome, %	74.6(1.3)	69.6(3)	0.1075
Current smoker, %	24.1(1.4)	23.5(2.8)	0.8472
Drinker, %	52.6(1.5)	48.7(3)	0.2551
Regular exercise (yes), %	22(1.3)	15.8(2.2)	0.0218^*^
Higher education, %	46.8(1.6)	40.3(3.2)	0.0658
Low income, %	26.7(1.3)	29.5(2.8)	0.3607
DR stage, %			
mild-to-moderate NPDR		80.5(2.4)	
severe NPDR		6.0(1.5)	
PDR		13.5(2.0)	

Data are presented as the mean ± standard error (SE) or proportions (SE). ^*^P < 0.05. BMI, body mass index; HbA1c, glycated hemoglobin; FPG, fasting plasma glucose; GFR, glomerular filtration; DR, diabetic retinopathy; NPDR, nonproliferative diabetic retinopathy; PDR, proliferative diabetic retinopathy.

### The relationship between hemoglobin levels and DR

The hemoglobin levels of patients with and without DR were compared using four different models (Table 2). Model 1 was not adjusted for any covariates. Model 2 was adjusted for age and sex. Model 3 was adjusted for age, sex, BMI, smoking, drinking, exercise, education, and income. Model 4 was adjusted for age, sex, BMI, smoking, drinking, exercise, education, income, DM duration, insulin therapy, HbA1c, and eGFR. In Models 1, 2, and 3, the hemoglobin levels were significantly higher in patients without DR than in those with DR (*P* < 0.001). In Model 4, the hemoglobin levels differed based on DR status (*P* = 0.006).

**Table 2:** The comparison of hemoglobin levels by diabetic retinopathy status.

	No DR	DR	P value
Model 1	14.33 ± 0.05	13.76 ± 0.12	<0.0001
Model 2	14.13 ± 0.04	13.67 ± 0.1	<0.0001
Model 3	14.12 ± 0.04	13.67 ± 0.1	<0.0001
Model 4	14.1 ± 0.04	13.73 ± 0.10	0.0007

Model 1 is unadjusted.

Model 2 is adjusted for age and sex.

Model 3 is adjusted for age, sex, BMI, smoking, drinking, exercise, education, and income.

Model 4 is adjusted for age, sex, BMI, smoking, drinking, exercise, education, income, DM duration, Insulin therapy, HbA1c, and eGFR.

Table 3 shows the association between hemoglobin levels and DR. The DR risk decreased significantly with an increase in hemoglobin. In Model 1, a 19% decrease in DR risk was observed with a 1.0-g/dL increase in hemoglobin level [odds ratio (OR) = 0.813; 95% confidence interval (CI) = 0.746–0.886; *P* < 0.0001]. The results of Models 2, 3, and 4 were similar. Likewise, the DR risk decreased significantly with a 0.5-g/dL increase in hemoglobin level. After full model adjustment, a 0.5-g/dL increase in hemoglobin level was significantly associated with a decreased DR risk (OR = 0.899; 95% CI = 0.848–0.953; *P* = 0.0004).

**Table 3:** The relationship between hemoglobin levels and diabetic retinopathy.

	Model 1		Model 2		Model 3		Model 4	
	OR (95% CI)	P-value	OR (95% CI)	P-value	OR (95% CI)	P-value	OR (95% CI)	P-value
Hemoglobin of 1 g/dL	0.813 (0.746–0.886)	<0.0001	0.77 (0.694–0.853)	<0.0001	0.777 (0.696–0.867)	<0.0001	0.807 (0.718–0.907)	0.0003
Hemoglobin of 0.5 g/dL	0.902 (0.864-0.941)	<0.0001	0.877 (0.833–0.924)	<0.0001	0.881 (0.834–0.931)	<0.0001	0.899 (0.848–0.953)	0.0004

Model 1 is unadjusted.

Model 2 is adjusted for age and sex.

Model 3 is adjusted for age, sex, BMI, smoking, drinking, exercise, education, and income.

Model 4 is adjusted for age, sex, BMI, smoking, drinking, exercise, education, income, DM duration, insulin therapy, HbA1c, and eGFR.

### DR risk by quartiles of hemoglobin levels

Table 4 presents the risk of DR in four groups according to quartiles of hemoglobin levels (Q1–Q4). The mean hemoglobin levels were 12.0 ± 0.1 g/dL in Q1, 13.5 ± 0.01 g/dL in Q2, 14.5 ± 0.02 g/dL in Q3, and 16.2 ± 0.04 g/dL in Q4. The highest quartile of hemoglobin level (Q4) yielded a 62% decrease in the risk of DR compared to the lowest quartile (Q1) (OR = 0.386; 95% CI = 0.257–0.579). The DR risk was also decreased in Q2 (OR = 0.607; 95% CI = 0.417–0.882) and Q3 (OR = 0.731; 95% CI = 0.501–1.069) also decreased. There was a linear trend in the DR risk across hemoglobin quartiles. After full model adjustment, the risk of DR tended to decrease at higher hemoglobin quartiles (*P* for trend <0.0033). The adjusted OR of DR was lowest in the highest hemoglobin quartile (*P* = 0.0035). The patients were further divided into hemoglobin deciles to stratify the DR risk. The prevalence of DR and the adjusted OR of DR showed decreasing trends with increasing hemoglobin levels (*P* for trend <0.0001; Supplementary Table S1).

**Table 4:** The risk of diabetic retinopathy according to quartiles of hemoglobin levels.

	Hemoglobin quartiles				*P* for trend
	Q1 (n = 515)	Q2 (n = 531)	Q3 (n = 553)	Q4 (n = 524)	
Hemoglobin, g/dL	12.0 ± 0.1	13.5 ± 0.01	14.5 ± 0.02	16.2 ± 0.04	<0.0001
Model 1 OR (95% CI)	1 (ref.)	0.607 (0.417–0.882)	0.731 (0.501–1.069)	0.386 (0.257–0.579)	<0.0001
Model 2 OR (95% CI)	1 (ref.)	0.587 (0.404–0.854)	0.637 (0.425–0.955)	0.311 (0.188–0.514)	<0.0001
Model 3 OR (95% CI)	1 (ref.)	0.6 (0.409–0.88)	0.676 (0.445–1.026)	0.331 (0.194–0.563)	0.0001
Model 4 OR (95% CI)	1 (ref.)	0.746 (0.482–1.155)	0.880 (0.562–1.378)	0.392 (0.220–0.699)	0.0035

Model 1 is unadjusted.

Model 2 is adjusted for age and sex.

Model 3 is adjusted for age, sex, BMI, smoking, drinking, exercise, education, and income.

Model 4 is adjusted for age, sex, BMI, smoking, drinking, exercise, education, income, DM duration, insulin therapy, HbA1c, and eGFR.

### Hemoglobin levels and the prevalence of anemia at different stages of DR

**Figure 1:**
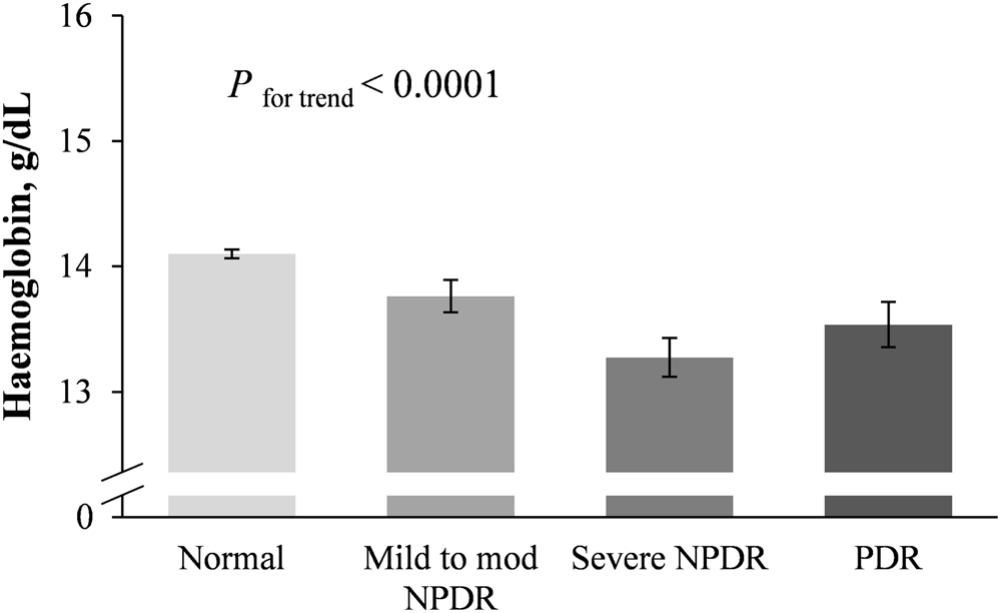
Hemoglobin levels at different stages of diabetic retinopathy. Hemoglobin levels showed a decreasing trend with more advanced stages of DR. *P* for trend <0.0001; Error bars indicate 95% confidence intervals; DR, diabetic retinopathy; NPDR, mild-to-moderate nonproliferative; PDR, proliferative diabetic retinopathy.

**Figure 2:**
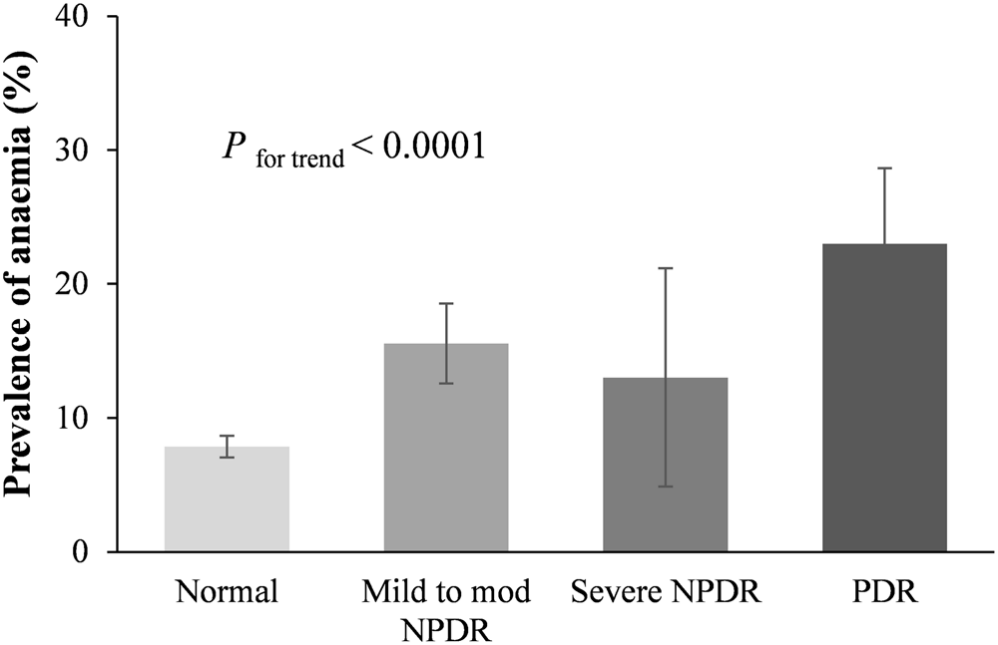
Prevalence of anemia at different stages of diabetic retinopathy. The prevalence of anemia showed an increasing trend with more advanced stages of DR. *P* for trend <0.0001; Error bars indicate 95% confidence intervals; DR, diabetic retinopathy; NPDR, mild-to-moderate nonproliferative; PDR, proliferative diabetic retinopathy.

Hemoglobin levels and the prevalence of anemia differed at different stages of DR (no DR, mild to moderate NPDR, severe NPDR, and PDR). The hemoglobin levels were 14.1 ± 0.1, 13.8 ± 0.1, 13.3 ± 0.2, and 13.5 ± 0.2 g/dL, from no DR to PDR, respectively and showed a significant decreasing trend at more advanced stages of DR (*P* for trend < 0.0001; Fig. 1). The hemoglobin levels were lower in severe NPDR than in PDR, but the difference was not significant. Intergroup comparisons revealed significant reductions in hemoglobin in the groups without DR vs. those with mild to moderate NPDR, severe NPDR, and PDR and in the groups with mild to moderate NPDR vs. those with severe NPDR. The prevalence of anemia was 7.8 (0.8), 15.5 (2.9), 13.1 (8.2), and 22.9 (5.6)%, from no DR to PDR, respectively and also showed a significant increasing trend with progressive severity of DR (*P* for trend < 0.0001; Fig. 2). The prevalence of anemia was higher in mild to moderate NPDR than in severe NPDR, but the difference was not significant. Intergroup comparisons revealed a significant rise in the prevalence of anemia in the mild to moderate NPDR, severe NPDR, and PDR groups compared with patients without DR.

## Discussion

In this study, patients with DR had lower hemoglobin levels and higher rates of anemia. High hemoglobin levels were significantly associated with a decreased risk of DR in patients with type 2 DM. The relationship between hemoglobin levels and DR was independent of age, sex, duration of DM, BMI, HbA1c, and eGFR. The risk of DR tended to decrease at higher quartiles of hemoglobin levels. Moreover, hemoglobin levels and the prevalence of anemia exhibited a linear trend with increasing DR severity.

Low hemoglobin levels and anemia have been associated with DR risk. Different factors have been associated with the development and severity of DR in patients with type 2 DM^17–19^. These include DM duration, HbA1c, high blood pressure, albuminuria, anemia, etc. In the present study, age, DM duration, BMI, FPG, HbA1c, eGFR, hemoglobin, and anemia differed by DR status. In addition, hemoglobin levels may vary not only by sex and age but also by nutrition, socioeconomic status, ethnicity, race, altitude and smoking status^20^. We accounted for variables that potentially affect the hemoglobin level. Model 3 was adjusted for age, sex, BMI, smoking, drinking, exercise and education level based on the statistical significance in the present study. Model 4 was further adjusted for DM duration, insulin therapy, HbA1c, and eGFR, which were implicated in the development of DR. After adjusting for these confounding factors, the hemoglobin levels were still significantly lower in patients with DR compared with those without DR. In addition, higher hemoglobin levels were associated with a lower DR risk. Even after adjusting for variables associated with hemoglobin and DR, the results persisted.

The relationship between hemoglobin levels and DR has been reported in several studies of type 2 diabetes^14–16^. One study suggested that decreased hemoglobin may cause direct organ damage as a possible mechanism responsible for this association^21^. The author hypothesized that low hemoglobin, through a reduction in shear stress, fosters the development of DR. In small vessels, shear stress is important in regulating the synthesis of nitric oxide and controlling vessel tone and angiogenesis^22^. In the retina, shear stress may also influence the function and activity of the retinal microvessels, acting on endothelial cells and pericytes, which are important regulators of vascular remodeling and tone. The dysfunction and loss of these cells promotes the development or worsening of DR^23^. Another study showed that hemoglobin levels were inversely related with endothelial function in patients with DM^24^. In this study, hemoglobin levels displayed a decreasing trend with progressive severity of DR, suggesting their role in mechanisms leading to DR progression.

Whether anemia contributes directly to the acceleration of complications in DR remains uncertain; hence, anemia is considered an independent risk factor for the development and progression of DR in patients with type 2 DM^13^. Our results show an increased prevalence of anemia in patients with more advanced stages of DR. It is well established that DR is associated with tissue hypoxia, as a result of impaired autoregulation of the microvasculature and capillary occlusion^25^. Tissue hypoxia and ischemia are crucial components of DR, which is potentially influenced by anemia^26^. Studies have indicated that anemic diabetic patients have elevated levels of vascular endothelial growth factor (VEGF), a marker of tissue ischemia. A potent stimulant of neovascularization also enhances capillary permeability, resulting in tissue edema and retinal exudates^27^. Hence, anemia is thought to exacerbate the progression of retinal ischemia in patients with DR^28^. Consistent with this hypothesis, several small studies have demonstrated that treating anemia in patients with DM may be associated with reductions in tissue edema and retinal exudates^29,30^.

Tissue hypoxia is a critical component of DR, and it is potentially influenced by anemia, the main indicator of blood oxygen delivery capacity^12^. This suggests that hypoxia stimulates the release of inflammatory mediators and vasoproliferative factors, including VEGF and erythropoietin (EPO), which are capable of increasing vascular permeability and contribute to the development of macular edema and more severe forms of DR. Studies have also shown that *VEGF* mRNA expression levels are significantly elevated in the retinas of diabetic rats, primarily in the retinal ganglion cell layer and the inner nuclear layer^31^. High intravitreal levels of EPO have been observed in cases of proliferative DR and in patients with diabetic macular edema^32,33^. Hypoxia decreases adenosine triphosphate levels and induces ion imbalances and free radicals, which cause the apoptosis of ganglion cells, inner nuclear layer cells, and retinal pigment epithelium cells^34^.

The association between hemoglobin levels and retinopathy is well known. The effect of low hemoglobin levels on fundus lesions is not completely understood, but seems to be related to retinal hypoxia^35^. Previous studies have indicated that anemia may be associated with retinal lesions^36^. Our results show that hemoglobin levels were associated with DR risk in type 2 diabetic patients. An adequate supply of oxygen and nutrients is critical for retinal function. Prolonged hypoxia induced by high glucose levels can induce changes in the retina. Retinal hypoxia that occurs at low hemoglobin or high glucose levels can lead to cellular damage at the molecular level.

Anemia is a common complication in patients with type 2 DM^37^ and is associated with disease progression and the development of comorbidities^38^. Diabetic kidney disease is clearly a major cause of anemia in patients with DM^7^; however, an increased risk of anemia is also observed in patients without renal impairment^39^. Anemia has been associated with the development and progression of diabetic vascular complications; however, its direct role in the development or progression of DR remains to be clearly established. Moreover, the clinical utility of correcting anemia in patients with DR has yet to be demonstrated in randomized controlled trials. In this study, we demonstrated that increased hemoglobin levels were independently associated with a decreased risk of DR. In addition, we observed a significantly negative relationship between hemoglobin levels and DR stage. Further study is required to confirm whether increased hemoglobin levels improve DR.

Although the current study did not show effects of altitude on DR, the average altitude of Korea is 482 m, lower than the world average (approximately 875 m). At higher altitudes (>1,000 m above sea level), the partial pressure of oxygen is decreased in the atmosphere, resulting in lower oxygen saturation of the blood^40^. At high altitudes, hypoxia induces vascular engorgement, retinal hemorrhage, and vascular tortuosity. High altitude retinopathy and the vascular proliferation of DR are responses to different types of tissue hypoxia, and there may be a synergistic effect between the two conditions. However, whether high altitude contributes to or aggravates retinal microangiopathy in diabetes is unknown^41,42^. Additional studies are needed to assess the effects of high altitude on patients with DR.

We found that BMI and waist circumference were significantly higher in patients without DR than in those with DR. Multiple studies have reported the effects of BMI on DR risk, but the results remain controversial^43^. To date, few mechanisms can account for the neutral association between BMI and DR. It is assumed that obesity has both protective and adverse effects on DR risk. Additional studies are needed to confirm these findings. Moreover, there were no significant differences in total cholesterol, hypertension, or metabolic syndrome between the two groups.

We performed a cross-sectional study using Korea National Health and Nutrition Examination Survey (KNHANES) data. This general population-based data set could reduce the potential selection bias of using hospital data. However, this study has several limitations. First, the results do not provide information on potential causative effects, owing to the study design. Second, hemoglobin levels were measured with only a single blood sample. No information on the history of anemia or specific drug use, which may affect hemoglobin levels, was available; thus, anemia was determined based on the World Health Organization’s definition using hemoglobin levels, and should be interpreted with caution. Third, although we adjusted for potential confounding factors, we cannot exclude the possibility that DR was affected by other variables. Additional cohort studies and intervention trials should be conducted to establish a relationship between hemoglobin levels and DR. Finally, there were differences in the subjects at each DR stage, and we presented the *P*-value for the trend, which indicates not the group difference but the group tendency, rather than the *P*-value. In addition, the classification of retinopathy was based on non-mydriatic photographs, and important lesions may have been missed. Thus, a well-designed randomized controlled trial is required to verify these results.

In conclusion, we found that high hemoglobin levels were significantly related to a decreased DR risk in Korean patients with type 2 diabetes. Moreover, hemoglobin levels showed a decreasing trend with DR severity. These findings have clinical implications for the potential benefits of better control of hemoglobin levels in patients with DR. Additional clinical trials are needed to clarify the causal relationship between hemoglobin levels and DR.

## Methods

### Study subjects

This study examined data from the KNHANES 2008–2012, the second and third year of the KNHANES IV as well as the KNHANES V. A total of 25,839 individuals older than 30 years was included in this study. DM was defined by an FPG level ≥126 mg/dL, the use of insulin or oral hypoglycaemic agents, or diagnosis by a self-reported history of physician diagnosis. A total of 23,160 participants who did not have DM and 556 who had missing data related to DM were excluded. Ultimately, 2,123 participants (1,065 men and 1,058 women) with DM were included in the analysis.

The KNHANES is a nationwide, population-based, representative cross-sectional survey conducted annually by the Korea Centers for Disease Control and Prevention (KCDC). The data from KNHANES provide statistical information for health-related policies in Korea, which also serve as the research infrastructure for studies on risk factors and diseases^44^. This survey was approved by the KCDC institutional review board and adhered to the tenets of the Declaration of Helsinki for biomedical research. All participants signed an informed consent form.

### Clinical information and laboratory analysis

The survey collected information on anthropometric measures, biochemical and clinical profiles of non-communicable diseases, healthcare utilization, health-related behaviors, and socioeconomic status. Blood and urine samples were obtained in the morning after overnight fasting. HbA1c was measured using an HLC-723G7 automated glycohemoglobin analyzer (Tosoh, Tokyo, Japan), and FPG, cholesterol, and hemoglobin were measured using a Hitachi 7600 automatic analyser (Hitachi Ltd., Tokyo, Japan). The eGFR was calculated using an equation derived from the Modification of Diet in Renal Disease study^45^. We used the definitions of anemia given by the National Anemia Action Council and the World Health Organization for simplicity.

### Ophthalmic examination and definition of DR

Participants underwent ocular examinations, including stereofundus photography. A 45° non-mydriatic digital retinal image centered on the fovea was taken of each eye, for a total of two images per person^46^. A TRC-NW6S non-mydriatic fundus camera (Topcon, Tokyo, Japan) linked to a D-80 camera (Nikon, Tokyo, Japan) was used to obtain digital fundus images. Retinopathy was graded based on the presence of microaneurysms, haemorrhages, hard exudates, area of revascularization, fibrous proliferation, and/or laser scars in the more severely affected eye. The Early Treatment Diabetic Retinopathy Study scale was used to define the severity of DR: 1, absence of retinopathy (level 10); 2, mild to moderate NPDR (level 20, 35, and 43); and 3, severe NPDR and proliferative DR (level ≥47)^47^. The classification of DR was described in a previous study^48^. The quality of the survey was verified by the Epidemiologic Survey Committee of the Korean Ophthalmological Society.

### Statistical analysis

All analyses were performed using the Statistical Analysis System statistical software package, version 9.3 (SAS Institute Inc., Cary, NC, USA). The KNHANES is a complex, stratified, multistage, probability-cluster survey that uses multiple complex survey designs^49^. Patient characteristics were compared using the *t*-test for continuous variables and chi-square test for categorical variables. Data are presented as the mean ± standard error (SE) for continuous variables or proportion (SE) for categorical variables. An analysis of covariance (ANCOVA) was used to determine the differences in hemoglobin levels in four different models. Model 1 was unadjusted; Model 2 was adjusted for age and sex; Model 3 was adjusted for age, sex, BMI, smoking, drinking, exercise, education, and income; and Model 4 was adjusted for age, sex, BMI, smoking, drinking, exercise, education, income, DM duration, insulin therapy, HbA1c, and eGFR. A multiple logistic regression analysis was performed to examine the association between hemoglobin levels and DR in four different models. ORs were calculated to determine the DR risk with increased hemoglobin. A linear trend of hemoglobin levels and the prevalence of anemia based on the stage of DR were analyzed using a general linear model. An ANCOVA was performed to assess differences between the groups. A two-sided *P*-value < 0.05 was considered statistically significant, and 95% CIs were calculated.

### Data availability

The data that support the findings of this study are available from the Korea Centers for Disease Control and Prevention database through the following http://cdc.go.kr/CDC/contents/CdcKrContentView.jsp?cid=60599&menuIds=HOME001-MNU1130-MNU1639-MNU1640-MNU1642,http://cdc.go.kr/CDC/contents/CdcKrContentView.jsp?cid=60940&menuIds=HOME001MNU1130-MNU1639-MNU1748-MNU1752, and https://knhanes.cdc.go.kr/knhanes/eng/. Anybody who signs up for membership can get raw data from the webpage. Unfortunately, the data downloads are only available on the Korean site. Kyungdo Han (hkd917@naver.com), the second author, can help provide the data.

## Electronic supplementary material

Supplementary Table S1
