# Supplementary material for: High hemoglobin levels are associated with decreased risk of diabetic retinopathy in Korean type 2 diabetes
Source: Sci Rep. 2018 Apr 3;8:5538. doi: 10.1038/s41598-018-23905-2 (PMC5882879; doi:10.1038/s41598-018-23905-2)
Supplement: Supplementary file 1 — Supplementary Table S1 [file 41598_2018_23905_MOESM1_ESM.pdf]

High haemoglobin levels are associated with decreased risk of diabetic retinopathy in Korean patients with type 2 diabetes

Min-Kyung Lee[1], Kyung-Do Han[2], Jae-Hyuk Lee[1], Seo-Young Sohn[1], Jee-Sun Jeong[3], Mee-Kyoung Kim[3], Ki-Hyun Baek[3], Ki-Ho Song[3], Hyuk-Sang Kwon[3*]

[1]Division of Endocrinology and Metabolism, Department of Internal Medicine, Seonam University College of Medicine, Myongji hospital, Gyeonggi-do, Republic of Korea

[2]Department of Medical Statistics, College of Medicine, The Catholic University of Korea, Seoul, Republic of Korea

[3]Division of Endocrinology and Metabolism, Department of Internal Medicine, Yeouido St. Mary's Hospital, College of Medicine, The Catholic University of Korea, Seoul, Republic of Korea

***Corresponding Author:** Hyuk-Sang Kwon

Division of Endocrinology and Metabolism, Department of Internal Medicine, Yeouido St. Mary's Hospital, College of Medicine, The Catholic University of Korea, 10, 63-ro, Yeongdeungpo-gu, Seoul, 07345, Republic of Korea

Tel: +82-2-3779-1039, Fax: +82-2-785-5655, E-mail: drkwon@catholic.ac.kr

	%(SE)	OR(95% CI)*
D1	32.6(4.1)	1 (ref.)
D2	18.8(3.5)	0.583(0.304,1.118)
D3	17.9(3)	0.435(0.23,0.82)
D4	16.6(2.9)	0.543(0.282,1.045)
D5	16.1(3)	0.604(0.319,1.145)
D6	19(3.5)	0.622(0.335,1.156)
D7	19.4(3.3)	0.633(0.325,1.235)
D8	14.4(3)	0.441(0.213,0.914)
D9	9.5(2.7)	0.242(0.099,0.592)
D10	14.3(2.7)	0.389(0.179,0.847)
P for trend	<0.0001	<0.0001

Supplementary Table S1. The risk of diabetic retinopathy by deciles of haemoglobin levels

*Full model adjusted
